# Improvement of Charcot-Marie-Tooth Phenotype with a Nanocomplex Treatment in Two Transgenic Models of CMT1A

**DOI:** 10.34133/bmr.0009

**Published:** 2024-03-28

**Authors:** Mohamed El Massry, Zeina Msheik, Tarek El Masri, Gautier MA Ndong Ntoutoume, Laetitia Vignaud, Laurence Richard, Emilie Pinault, Pierre-Antoine Faye, Frédérique Bregier, Pierre Marquet, Frédéric Favreau, Jean-Michel Vallat, Fabrice Billet, Vincent Sol, Franck Sturtz, Alexis Desmouliere

**Affiliations:** ^1^ NeurIT UR20218, Faculty of Medicine and Pharmacy, University of Limoges, Limoges, France.; ^2^ Department of Anatomy, Cell Biology & Physiological Sciences, Faculty of Medicine, American University of Beirut, Beirut, Lebanon.; ^3^ LABC*i*S UR22722, University of Limoges, F-87000 Limoges, France.; ^4^ Reference Center for Rare Peripheral Neuropathies, Department of Neurology, University Hospital of Limoges, Limoges, France.; ^5^ BISCEm (Biologie Intégrative Santé Chimie Environnement) Platform, US 42 Inserm/UAR 2015 CNRS, University of Limoges, Limoges, France.; ^6^ Department of Biochemistry, University Hospital of Limoges, Limoges, France.; ^7^ INSERM U1248 Pharmacology & Transplantation, CBRS, Faculty of Medicine and Pharmacy, University of Limoges, Limoges, France.; ^8^ Department of Pharmacology and Toxicology, CHU Limoges, Limoges, France.

## Abstract

Curcumin has been shown to exert beneficial effects in peripheral neuropathies. Despite its known biological activities, curcumin has unfavorable pharmacokinetics. Its instability has been linked to its failure in clinical trials of curcumin for the treatment of human pathologies. For this reason, we developed curcumin-loaded cyclodextrin/cellulose nanocrystals (NanoCur) to improve its pharmacokinetics. The present study aims to assess the potency of a low dose of NanoCur in 2 Charcot-Marie-Tooth disease type 1A (CMT1A) rodent models at different stages of the disease. The efficiency of NanoCur is also compared to that of Theracurmin (Thera), a commercially available curcumin formulation. The toxicity of a short-term and chronic exposure to the treatment is investigated both in vitro and in vivo, respectively. Furthermore, the entry route, the mechanism of action and the effect on the nerve phenotype are dissected in this study. Overall, the data support an improvement in sensorimotor functions, associated with amelioration in peripheral myelination in NanoCur-treated animals; an effect that was not evident in the Thera-treated group. That was combined with a high margin of safety both in vivo and in vitro. Furthermore, NanoCur appears to inhibit inflammatory pathways that normally include macrophage recruitment to the diseased nerve. This study shows that NanoCur shows therapeutic benefits with minimal systemic toxicity, suggesting that it is a potential therapeutic candidate for CMT1A and, possibly, for other neuropathies.

## Introduction

Charcot-Marie-Tooth 1A (CMT1A) is the most common inherited form of neuropathy, belonging to a family of sensorimotor disorders generally referred to as CMT [1]. It is characterized by an alteration in the gene for peripheral myelin protein 22 (PMP22), leading to a length-dependent degeneration of peripheral nerves, that manifests as reductions in both nerve conduction velocity and compound muscle action potential, secondary to demyelination and axonal loss, respectively [2]. The sensorimotor neuropathy of CMT1A mainly results in lower limb muscle atrophy and foot deformities (pes cavus), that later extend to the hands resulting in reduced tendon reflexes and distal sensory dysfunction [3,4]. Despite the high prevalence of CMT (1:2,500) with CMT1A being the most common form [5], CMT1A treatment strategies are still scarce and most interventions aim at controlling patient symptoms and improving patient quality of life.

Curcumin-based treatments have been used on a vast array of neuropathies and preliminary data have shown promising neuroprotective outcomes. For instance, local administration of curcumin, at a modest dose, close to a traumatic lesion of the sciatic nerve accelerated sciatic nerve functional recovery [6]. In the context of CMT, curcumin improved the pathological phenotype in several models of demyelinating CMT neuropathies [7–9]. Additionally, oral administration and intraperitoneal injections were tested on rats with diabetic peripheral neuropathy [10,11], or alcohol-induced neuropathy [12], with beneficial effects observed in the rats following curcumin treatment. These effects were mainly attributed to the antioxidant and anti-inflammatory properties of the compound [13,14]. Nevertheless, curcumin has several limitations that could hinder its therapeutic use, including very low solubility in aqueous medium (about 0.6 μg/ml) requiring the use of organic solvents for in vivo administration and low stability at neutral pH. In fact, more than 90% of curcumin is degraded within 30 min in vitro [15] and a minute concentration of curcumin (approximately 2 μg/ml) was found in the plasma of patients within 2 to 3 h after ingestion of 10 to 12 g of curcumin [16].

We have previously reported that a novel curcumin nanocomplex (NanoCur) composed of cellulose nanocrystals (CNCs) bound to β-cyclodextrin (CD) overcomes the problems of weak solubility and poor pharmacokinetics of curcumin [17]. More importantly, an initial study showed a positive outcome associated with low-dose NanoCur administration for 8 weeks in young CMT1A rats. Indeed, the treatment resulted in a substantial improvement in nerve conduction velocity and motor performance of these rats. This was accompanied by an improved myelin phenotype and improved oxidative and endoplasmic reticulum (ER) stress status within the sciatic nerve [18]. In the present study, we aimed to assess the efficacy of NanoCur at a more advanced stage of the disease, where the illness appears to be more neurologically debilitating. In order to test whether the therapeutic effect of NanoCur could also be achieved by an alternative curcumin treatment, we also compared the efficacy of our compound with that of another commercially available curcumin formulation. For this purpose, Thera, a dried colloidal suspension containing 10% curcuminoids [19], was chosen. This compound has been shown to have the highest 0- to 24-h AUC (area under the curve) and relative bioavailability of curcuminoids in the systemic circulation among the available formulations of curcumin [20]. We also examined the efficacy of NanoCur in another transgenic model of disease, C61 mice, that presents with variable disease severity ranging from mild to severe. Lastly, we aimed to investigate, in depth, the mechanism of action of NanoCur, with a particular emphasis on inflammation, and the mechanism of curcumin-delivery into the cell. Our study also looked to confirm the lack of acute and long-term toxicity of curcumin following chronic treatment. This study paves the way for the potential employment of NanoCur in various neuropathies that share similar mechanisms of injury to CMT1A.

## Materials and Methods

### Experimental design

**Fig. 1.**
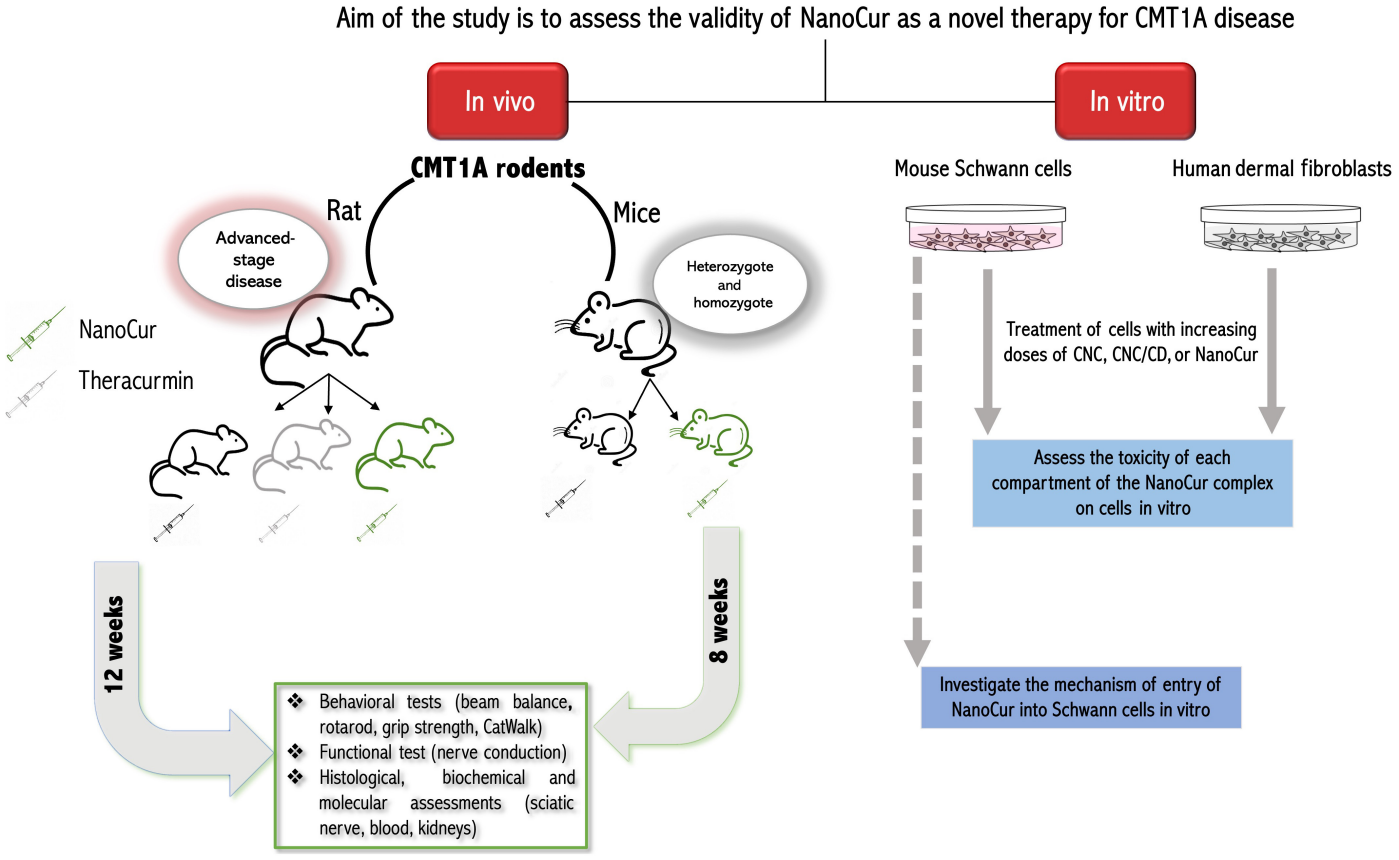
Diagram representing the experimental design employed for NanoCur validity testing in the current study.

The following study aims at investigating the efficiency of NanoCur as a potential new therapy for CMT1A disease. Hence in this study, 2 animal models were employed. CMT1A rats and mice were treated with NanoCur, and the behavioral, functional, and molecular outcomes were measured at the end of the treatment period (Fig. 1). In addition to testing the efficiency of the treatment with NanoCur in comparison to Theracurmin, a commercially available curcumin formulation, NanoCur toxicity as well as mechanism of entry and action were investigated in this study.

### Cells lines

An immortalized mouse Schwann cell model (MSC80) which can proliferate and express markers of myelination [21], and primary human dermal fibroblasts (HDF), were used. Cells were grown in complete Dulbecco’s modified Eagle’s medium containing glucose, 10% fetal bovine serum, 1% Glutamax, 1% nonessential amino acids and 1% penicillin/streptomycin. Similar to primary Schwann cells, MSC80 cells allow the dynamics of NanoCur to be studied in cells that are a model of the primary site of injury in CMT1A. However, MSC80s maintain the ability to proliferate, a characteristic that is lost upon Schwann cell maturation. HDF were also used to test the effects of NanoCur on fibroblasts exposed to NanoCur via systemic injection.

### In vitro toxicity

MSC80 and HDF cells were seeded in 6-well plates for 24 h. CNC and CNC/CD safety were assessed in MSC80 cells using an MTT assay (Abcam) after 24 and 48 h of treatment, at increasing concentrations from 10^-4^ mg/ml (previously determined as C_safe_) to 1 mg/ml. The live/dead assay was performed on both cell types using flow cytometry (CytoFLEX LX cytometer) in accordance with the manufacturer’s instructions (Fisher Scientific, L3224). The lethal dose 50 (LD50) was determined using GraphPad (Prism).

### Calcofluor white endocytosis assay

Endocytosis was tested in MSC80 cells using the calcofluor white (CW) assay. CW is a fluorescent dye known to bind with high affinity to cellulose. Briefly, cells were incubated for 2 h with both CNC/CD and CW in the presence or absence of endocytosis inhibitors dynasore (dynamin-mediated endocytosis inhibitor; 20 μM), or genestein (caveolae-mediated endocytosis inhibitor; 50 μM). Cells were then washed with Hanks’ balanced salt solution and incubated with a basic buffer (KOH+Hanks’ balanced salt solution) that promotes cell detachment and allows the release of intracellular fluorescent complexes. The entry of CW/CNC/CD complex into the cell was assessed by the relative fluorescence intensity using a Fluoroskan plate reader (Ex/Em = 355/433 nm).

### Animal models

The CMT1A transgenic rat model was used in the study [22]. Heterozygote rats carry 3 additional copies of the murine PMP22 gene, while homozygotes have 6 copies of the gene. Only heterozygote rats were employed due to the severe phenotype displayed by the homozygote rats. Male and female 3-month-old rodents weighing between 200 and 300 g were used. Rats were randomly divided into 6 groups and had daily intraperitoneal injections of either saline or 0.2 mg of curcumin/kg of body weight (either in Thera or NanoCur), a dose that was previously determined to be effective in young CMT1A rats [18], for a total of 12 weeks (Table 1).

**Table 1.** Experimental groups and number of rats per group used in the study

Animal type	Treatment	Number (*N*)
Wild-type (WT)	Saline	18
	Thera	9
	NanoCur	9
CMT1A (CMT)	Saline	13
	Thera	7
	NanoCur	16

In addition to rats, we used the C61 mouse model [23]. C61 mice bears 4 additional copies of the human PMP22 gene incorporated into their genome for the heterozygote mice (Tg^+/-^), while homozygotes (Tg^+/+^) present with 8 copies, making the phenotype more severe. In order to check the extent of NanoCur efficacy, both homozygote and heterozygote male and female mice were used. Unlike the rat model, due to the phenotypic and metabolic differences with the mouse model, 4-week-old mice were randomly divided and treatment with either saline or NanoCur was administered daily intraperitoneally at 0.4 mg of curcumin/kg of body weight, for a total period of 8 weeks (Table 2). All the animals were kept in cages that provided an enhanced habitat, a 12-h light/dark cycle, room temperature of 22 °C, and ad libitum access to food and water. Weights were checked once a week.

**Table 2.** Different experimental groups and number of mice per group used in the study

Mice	Treatment	Number (*N*)
Wild-type (WT)	Saline	5
	NanoCur	7
Heterozygote C61 (Tg^+/-^)	Saline	4
	NanoCur	5
Homozygote C61 (Tg^+/+^)	Saline	3
	NanoCur	4

The principles of laboratory animal care (NIH publication No. 86-23, revised 1985) were followed throughout the animal study, and all protocols were approved by the Regional Animal Experimentation Ethics Committee (CREEAL n° 16-2013-16) (APAFIS# 29437-2021020115213118 v1).

### Genotyping

Animals were genotyped using quantitative reverse transcription-polymerase chain reaction analysis of genomic DNA samples extracted from tail biopsies using the DNeasy blood and tissue kit according to the manufacturer’s instructions (Qiagen, Hilden, Germany). The presence of the PMP22 transgenes was detected by quantitative reverse transcription-polymerase chain reaction using mouse (5’-GTTCCTGTTCTTCTGCCAGC-3’;3’-CCTCATTCGCGTTTCCGCA-5’) or human (5’-TCAGGATATCTATCTGATTCTC-3’;3’- AAGCTCATGGAGCACAAAACC-5’)-specific PMP22 primers.

### Electrophysiology

Nerve conduction velocity was measured in the sciatic nerve of the experimental animals as previously described [18] using the PowerLab/26T (AD Instruments, Paris, France) after stimulating the sciatic nerve proximally and distally with supramaximal impulses. The latency difference between the sequential distal and proximal stimulations was used to calculate the motor nerve conduction velocity (MNCV).

### Beam balance test

A beam balance test was used to assess sensorimotor coordination in rodents as previously described in [18]. In brief, the rats were placed at the center of a metal rod and the falling latency was measured. For each rat, 3 trials, with a 5-min gap, were recorded.

### Gait analysis

The position of the hind feet during locomotion is entirely reliant on effective sciatic nerve function [24]. Using CatWalk XT Version 10.6 (Noldus Information Technology), we measured the toe spread between toes 1 and 5 of the hind limbs in rats. Average toe spread of each animal was calculated and plotted.

### Grip strength

A grip strength test was conducted to measure the neuromuscular force in our models as previously described in [18]. For each animal, 4 trials were carried out at 5-min intervals, and the mean of these tests was calculated as each animal’s grip strength.

### Accelerating rotarod

Mice were placed on the rotarod (Bioseb) to measure the latency and speed at which they fell within a 2-min interval while the speed of rotation was increased from 4 to 40 rpm. Three trials were performed for each animal, separated by 5 min of rest.

### Tissue extraction and biochemical tests

Rats were placed in metabolic cages for 24 h prior to euthanasia, and urine samples were collected. A Bradford assay was used, according to manufacturer’s instructions, to quantify proteinuria. The kidneys were removed and weighed to measure kidney hypertrophy. Additionally, blood was drawn, and creatinine, lactate dehydrogenase (LDH), creatine phosphokinase (CPK), aspartate transaminase (AST), and alanine transaminase (ALT) levels were measured by the clinical biochemistry platform of the Limoges University Hospital Center (CHU).

### Electron microscopy and morphometric analysis

Sciatic nerves were dissected from both rats and mice and embedded in epoxy resin after being fixed in 2.5% glutaraldehyde. Semithin (Nikon optical light microscope) and thin transverse sections (JEOL 1011 electron microscope) were used for morphometric analyses and g-ratio calculation (axon/fiber diameter), respectively, which were carried out by double-blinded examiners using random images (5 images, 100 axons/section).

### Macrophage detection in sciatic nerves

The sciatic nerves of rats were stained with rat anti-CD68 (ED1) primary antibody (Biorad, MCA341, 1:1,000) using the avidin-biotin 3,3-diaminobenzidine peroxidase kit (Vectastain; Vector Laboratories) in accordance with the supplier’s protocol and counterstained with Mayer’s hematoxylin, then observed using a Nikon optical microscope. Using ImageJ software (NIH, USA), the percent area of macrophages was quantified in 3 separate images per sample. For F4/80 (rat, 1:300, MCAP497, Serotec) staining of mouse tissue, immunofluorescence was performed as described in [25] and fluorescence visualized using a Zeiss LSM900 confocal microscope.

### Western blot

Sciatic nerves were dissected from wild-type (WT) and CMT1A rats and samples were processed for western blotting as described in [18], using antibodies against macrophage migration inhibitory factor (MIF) (1:2,000; Invitrogen, PA1-28840) and myeloperoxidase (1:2,000, ProteinTech, 102669-I-AP). Protein quantification was performed using ImageJ. Total proteins per lane were determined using stain-free capture of the membrane using the Chemidoc machine (Bio-Rad).

### Statistical analysis

All data are presented as mean + SEM (standard error of the mean). Normality (Shapiro–Wilk) and equality of variance (Brown–Forsythe) tests were first performed. The data were then compared using either a Student *t* test or a 1-way or 2-way analysis of variance (ANOVA) with repeated measurements, followed by a post hoc Tukey’s test. GraphPad statistical software was used to conduct all statistical analyses (GraphPad Software v.8, Inc., La Jolla, CA, USA). Differences were deemed significant when *P* < 0.05.

## Results

### Neurofunctional and behavioral benefits of NanoCur treatment in advanced stage CMT1A rats

**Fig. 2.**
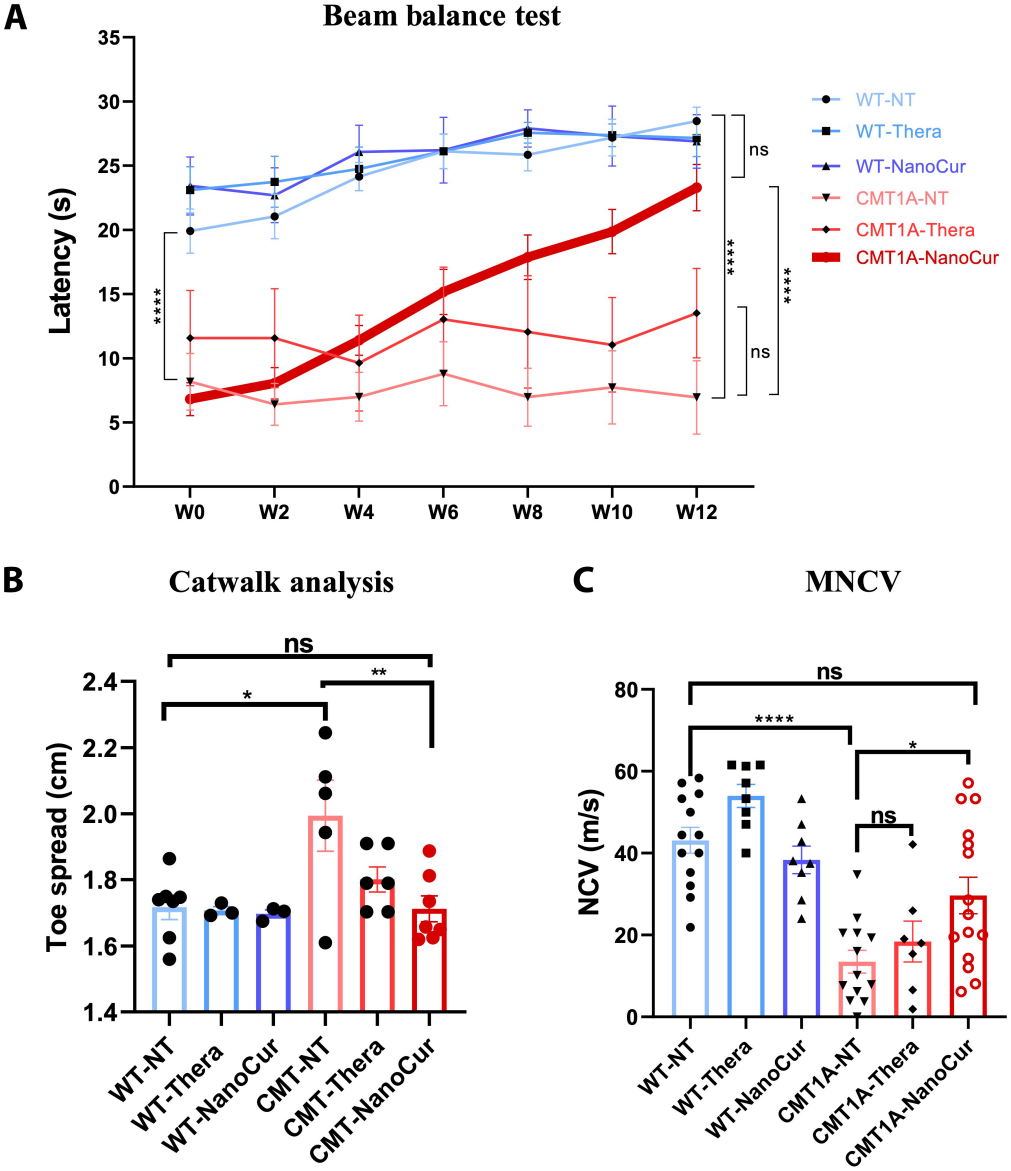
Neurofunctional and behavioral effect of NanoCur and Thera treatment on advanced stage CMT1A rats. Graph representing the evolution of sensorimotor coordination assessed periodically every 2 weeks in rats using the (A) beam balance test. (B) Catwalk/toe spread test was performed at the end of the treatment period as a measure of sensorimotor deficits. (C) The average MNCV was additionally measured in the experimental groups at week 12 immediately prior to euthanasia. Results are represented as mean ± SEM. Statistical test was performed using 1-way ANOVA followed by Tukey’s post hoc test for experiments including one variable (CatWalk and MNCV), or 2-way ANOVA followed by Tukey’s post hoc test for the experiments including 2 variables; ****: *P* < 0.0001, ***: *P* < 0.001 *: *P* < 0.05 (*n* = 18 WT-NT, *n* = 9 WT-Thera, *n* = 9 WT-NanoCur, *n* = 13 CMT1A-NT, *n* = 7 CMT1A-Thera and *n* = 16 CMT1A-NanoCur)

An initial study by Caillaud et al. [18] highlighted the therapeutic benefits of NanoCur treatment in early-stage CMT1A rats. Hence, we aimed to examine the therapeutic potential for NanoCur in a more advanced stage of the disease and compare it to that of another formulation, Thera. Firstly, the beam balance test showed a significant decrease in the latency to fall from the elevated rod in the CMT-saline group. Over the course of the treatment, the test revealed a progressive, significant improvement in equilibrium performance in CMT1A rats treated with NanoCur. However, this effect was not seen in the Thera-treated groups (Fig. 2A). The Catwalk gait test was also used to assess the sensorimotor function and revealed a discrepancy in the toe spread, where CMT1A rats had a larger spread between their first and fifth toes when walking, highlighting a decrease in muscle tonicity. The toe spread decreased to near control values when rats were treated with NanoCur. Thera treatment had a positive effect on toe spread but was less effective than NanoCur (Fig. 2B). MNCV was evaluated, and electrophysiological measurements showed a significantly reduced conduction speed (~ 3.3 fold) in untreated CMT1A compared to control rats. Treatment with Thera resulted in a slight improvement in MNCV, while NanoCur treatment resulted in a significant improvement in conduction velocity (~ 2.2 fold) when compared to the nontreated group (Fig. 2C).

### Association between NanoCur administration and an overall improvement in nerve histology of CMT1A rats

**Fig. 3.**
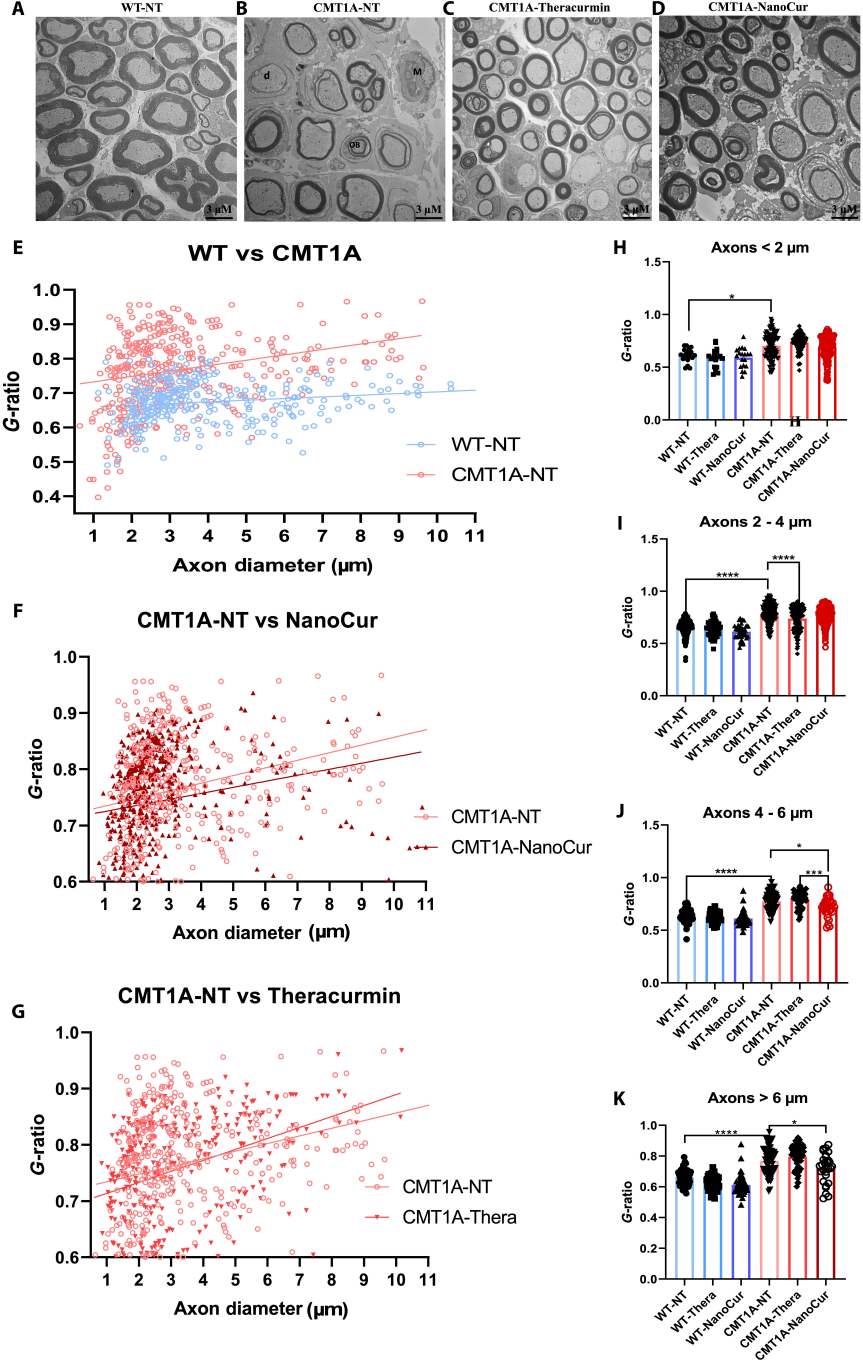
Histological assessments of the sciatic nerve in rat experimental groups. Representative images of the sciatic nerve morphology as assessed by EM (magnification 3,000x) in (A) WT-NT, (B) CMT1A-NT, (C) CMT1A-Thera, and (D) CMT1A-NanoCur rats. Demyelinated axon (d), macrophage (M), and onion bulb (OB) were marked in the CMT1A-NT nerves. *g*-ratio was plotted against axon diameter in WT versus CMT1A nontreated group (E), CMT1A nontreated and CMT1A-NanoCur treated (F), and CMT1A nontreated and Thera-treated rats (G); linear regression line is shown in the 3 graphs. Histograms representing the average *g-*ratio difference among the 6 rat groups (5 pictures, 100 axons/section), according to their caliber: (H) less than 2 μm, (I) between 2 and 4 μm, (J) between 4 and 6 μm, and (K) >6 μm. Results are represented as mean ± SEM. Statistical analysis was performed using 1-way ANOVA test followed by post hoc Tukey test; *: *P* < 0.05; **; *P* < 0.01; ****: *P* < 0.0001; WT-NT (*n* = 6), WT-Thera (*n* = 3), WT-NanoCur (*n* = 3), CMT1A-NT (*n* = 6), CMT1A- Thera (*n* = 5), and CMT1A-NanoCur (*n* = 7).

To confirm that the improvement in functional and behavioral parameters in the NanoCur-treated CMT1A rats was associated with changes at the histological level, sciatic nerves were examined by electron microscopy (EM). EM demonstrated that the nerves of CMT1A rodents are characterized by an increased number of demyelinated axons, the formation of onion bulbs (OBs), and macrophage (M) infiltration. Interestingly, NanoCur, but not Thera, significantly reversed this pathological phenotype (Fig. 3A to D). Semithin sections were also examined to provide a more accurate representation of the level of demyelination under different experimental conditions. A plot of *g*-ratio versus axon diameter showed that, for axons of similar diameter, CMT1A nontreated rats showed higher *g*-ratios with a higher linear regression plot, indicating a demyelination within the nerve (Fig. 3E). Treatment with NanoCur led to a decrease in the *g*-ratio, while treatment with Thera had no effect (Fig. 3F and G). When the *g*-ratio was further subdivided according to the axonal diameter, we observed a significant increase in the g-ratio in the CMT1A-NT group in all axon categories: axons smaller than 2 μm, between 2 and 4 μm, between 4 and 6 μm, and large axons that are greater than 6 μm. The examination of *g*-ratio according to axon diameter revealed a significant effect of NanoCur on medium and large caliber axons, with a lesser effect on the lower caliber axons. Conversely, Thera failed to show an effect on larger caliber axons but improved the myelin status in the 2- to 4-μm axons (Fig. 3H to K).

### Validation of NanoCur as a potential treatment for CMT1A in another transgenic model, C61 mice

**Fig. 4.**
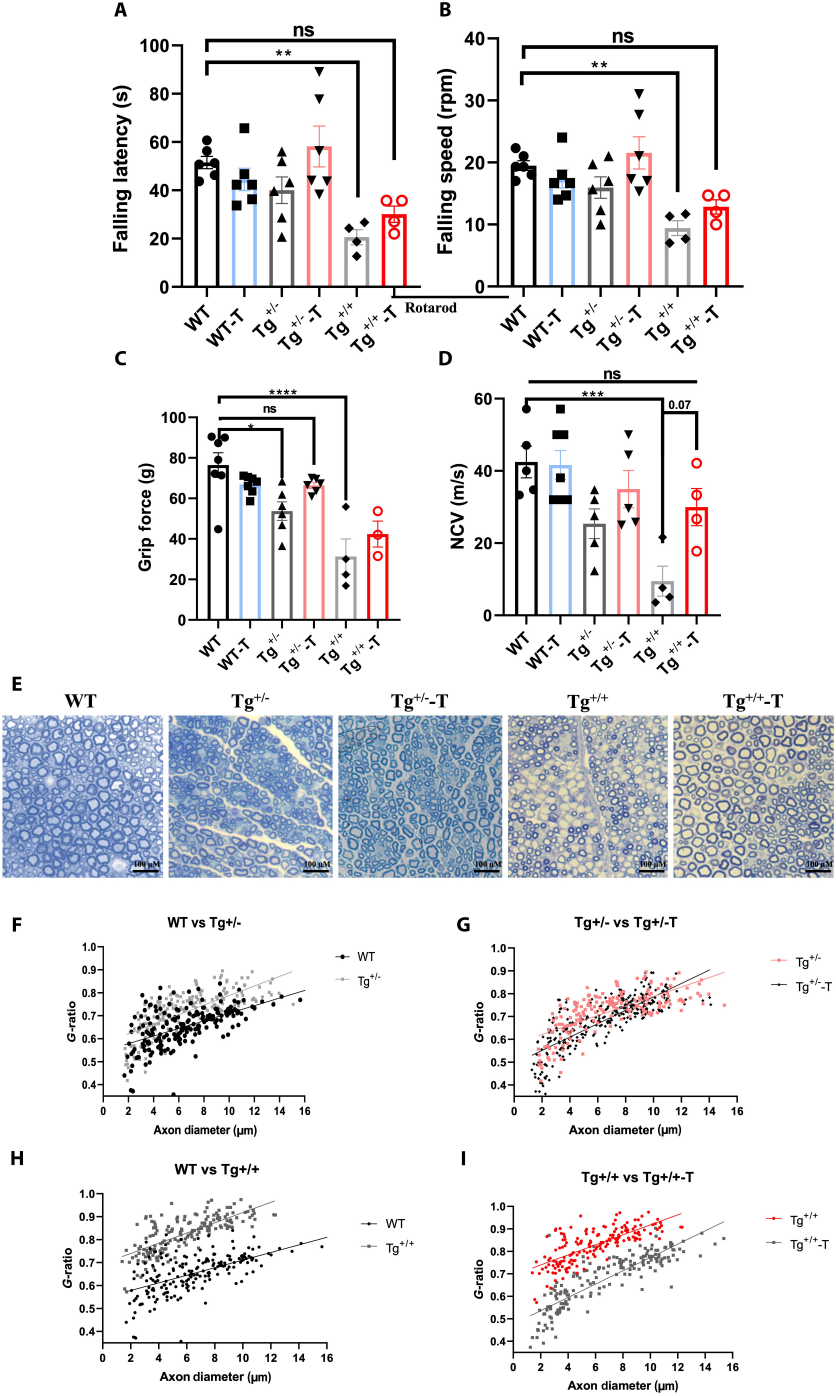
Validation of therapeutic benefits of NanoCur in C61 transgenic mice. Behavioral and functional tests carried out after the 8 weeks of treatment with NanoCur at a dose of 0.4 mg of Curcumin in NanoCur/kg of body weight: (A) Falling latency and (B) speed using the accelerating rotarod test, (C) hind paw grip, and (D) MNCV. (E) Toluidine Blue staining was also performed on the sciatic nerves to visualize the level of demyelination in each of the experimental groups. *g*-ratio was also plotted against axon diameter for the sciatic nerve (5 pictures, 100 axons/section) with WT group compared to Tg^+/-^ (F) and Tg^+/+^ (G) first. Tg^+/-^ nontreated was then compared to Tg^+/-^ treated with NanoCur (H) and Tg^+/+^ nontreated to Tg^+/+^-NanoCur (I), with linear regression shown in all of the graphs. Results are represented as mean ± SEM. Statistical analysis was performed using 1-way ANOVA test followed by post hoc Tukey test; *: *P* < 0.05; **; *P* < 0.01; *P* < 0.01; ****: *P* < 0.0001; WT (*n* = 5), WT-NanoCur (*n* = 7), Tg^+/-^ (*n* = 4), Tg^+/-^-NanoCur (*n* = 5), Tg^+/+^ (*n* = 4), Tg^+/+^-NanoCur (*n* = 3 to 4).

To further validate the therapeutic potential of NanoCur, the efficacy of the compound was investigated in a first study in the C61 mouse model. Interest in this model is due to it being a heterogeneous population of animals, in which disease severity varies from moderate to a very severe phenotype. Furthermore, homozygote mice are characterized by an extreme phenotype that leads to paralysis of the animals and ultimately to death [23]. Following 8 weeks of daily intraperitoneal treatment with 0.4 mg/kg of curcumin as NanoCur, assessment of sensorimotor function using the accelerating rotarod showed a reduced latency to fall and a lower speed of movement for the heterozygote Tg^+/-^ mice compared to WT mice, while treatment with NanoCur showed an improvement in the motor performance of the affected mice. This effect was also observed, but to a lower extent, in homozygous mice, taking into account the greater motor dysfunction in these animals (Fig. 4A and B). In addition, nontreated Tg^+/-^ mice displayed a significant decline in grip force, even greater in the Tg^+/+^ group; an effect that appeared to be ameliorated with NanoCur injection (Fig. 4C). More importantly, treatment with NanoCur led to a 1.4-fold increase in MNCV in the treated Tg^+/-^ mice compared to nontreated littermates, and a more potent enhancement of MNCV in homozygote mice, equivalent to ~3 fold compared to nontreated littermates (Fig. 4D). Histologically, evaluation of the *g*-ratio against axonal diameter showed an increase in the *g*-ratio of the Tg^+/-^ mice, in which most of the axons were within the higher *g*-ratio compartment compared to axons of similar diameter in the WT mice. This difference was even more marked in the Tg^+/+^ group. Importantly, treatment with NanoCur led to a downward shift in this parameter in most of the axonal population, indicating an improvement in myelin phenotype in the diseased animals (Fig. 4E to I).

### NanoCur in CMT1A pathology, a triple hit mechanism involving anti-inflammatory effects

**Fig. 5.**
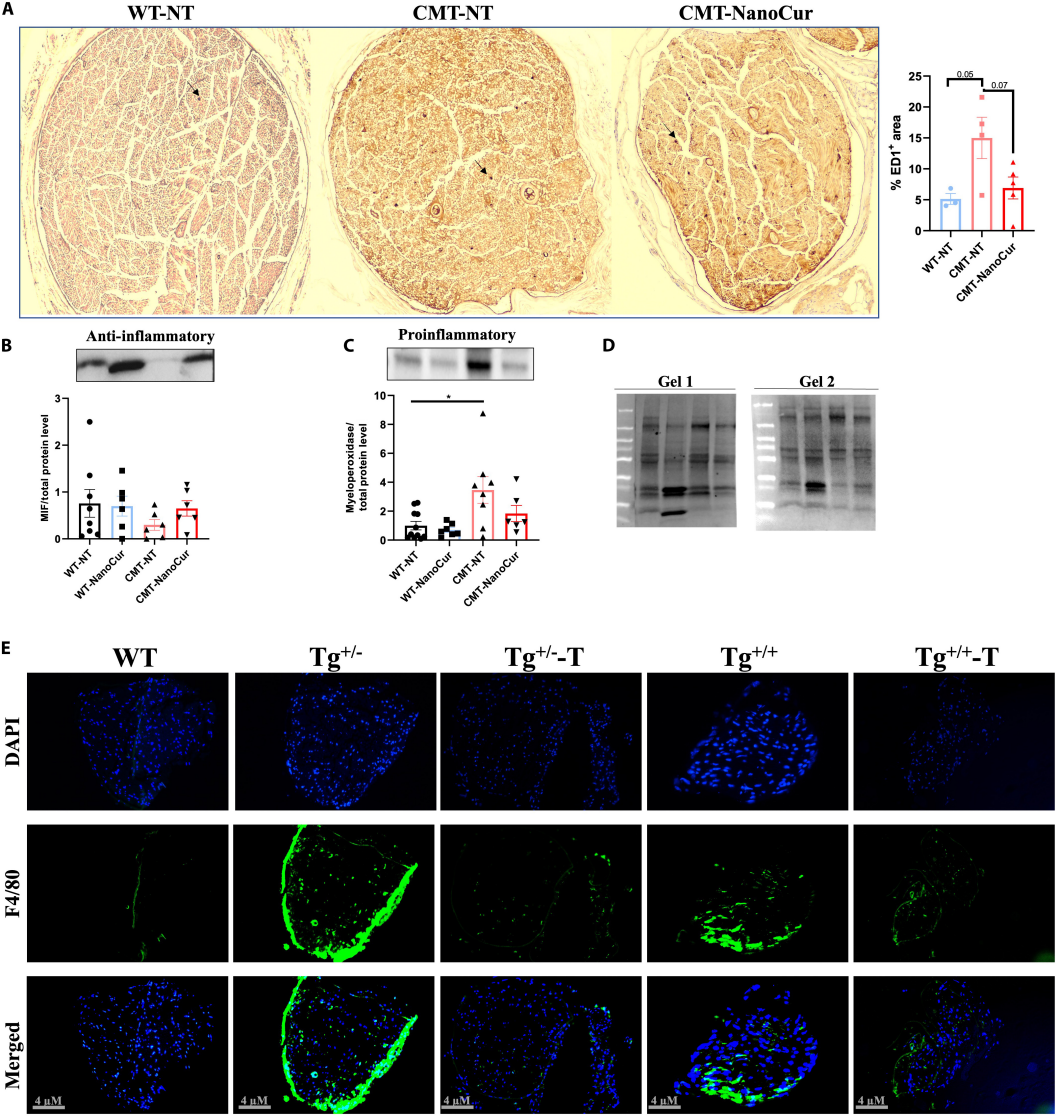
Investigation of the anti-inflammatory effect of NanoCur in CMT1A. (A) Anti-CD68 [ED1] staining of sciatic nerves using 3,3-diaminobenzidine staining (IHC) for macrophage detection in sciatic nerve sections of WT-NT, CMT1A-NT, and CMT1A-NanoCur rats, in addition to the quantification of the percentage area of macrophages present in the nerve sections. Representative figures along with barograms of the quantification of the average protein expression level of (B) Macrophage Migration Inhibition Factor MIF and (C) myeloperoxidase with respect to the total protein. (D) Stain-free gel of the representative figure for MIF (Gel 1) and myeloperoxidase (Gel 2) (E) Representative figure of the fluorescent staining of mouse sciatic nerves of WT, Tg^+/-^, Tg^+/-^-T, Tg^+/+^, and Tg^+/+^-T with the macrophage marker F4/80. Results are represented as mean ± SEM. Statistical analysis was performed using 1-way ANOVA test followed by post hoc Tukey test; *: *P* < 0.05.

Our previous work has shown that the beneficial effects of NanoCur occur via improvement in oxidative status in the sciatic nerve of CMT1A rats by activating the antioxidant defense system. In parallel, proteomics showed increased expression of ER stress response proteins, highlighting an increased activity in response to altered protein folding response, PMP22, in CMT1A rats [18]. These mechanisms were also examined in the older CMT1A rats and the results confirmed that NanoCur alleviated the ER stress response and oxidative stress through the correction of Calnexin and Superoxide dismutase 1 protein levels, respectively (data not shown). Given the anti-inflammatory effects linked to curcumin [26] and recent reports associating the development of CMT1A to an inflammatory process involving macrophages [25,27], we examined the effect of NanoCur on the inflammatory response in this model. ED-1 (anti-CD68) staining of sciatic nerves in CMT1A rats showed increased macrophage localization in the nerves of nontreated animals, while treatment with NanoCur led to reduced macrophage staining (Fig. 5A)*.* That was accompanied by an increase in the anti-inflammatory MIF protein and a decline in the proinflammatory myeloperoxidase (Fig. 5B to D and fig. S1). Macrophage recruitment was also investigated in the sciatic nerve of C61 mice by staining for F4/80. Increased F4/80 staining was observed in the Tg^+/-^ nerves, which was even more marked in the Tg^+/+^ untreated group. NanoCur significantly reduced macrophage-specific staining in both heterozygous and homozygous mice (Fig. 5E). These preliminary findings point NanoCur having effects on 3 pathways, antioxidant response, reducing inflammatory response and alleviating ER stress, potentially leading to correction of PMP22 levels, as observed in the previously published data in younger rats [18].

### Assessment of NanoCur cellular entry mechanism and toxicity

**Fig. 6.**
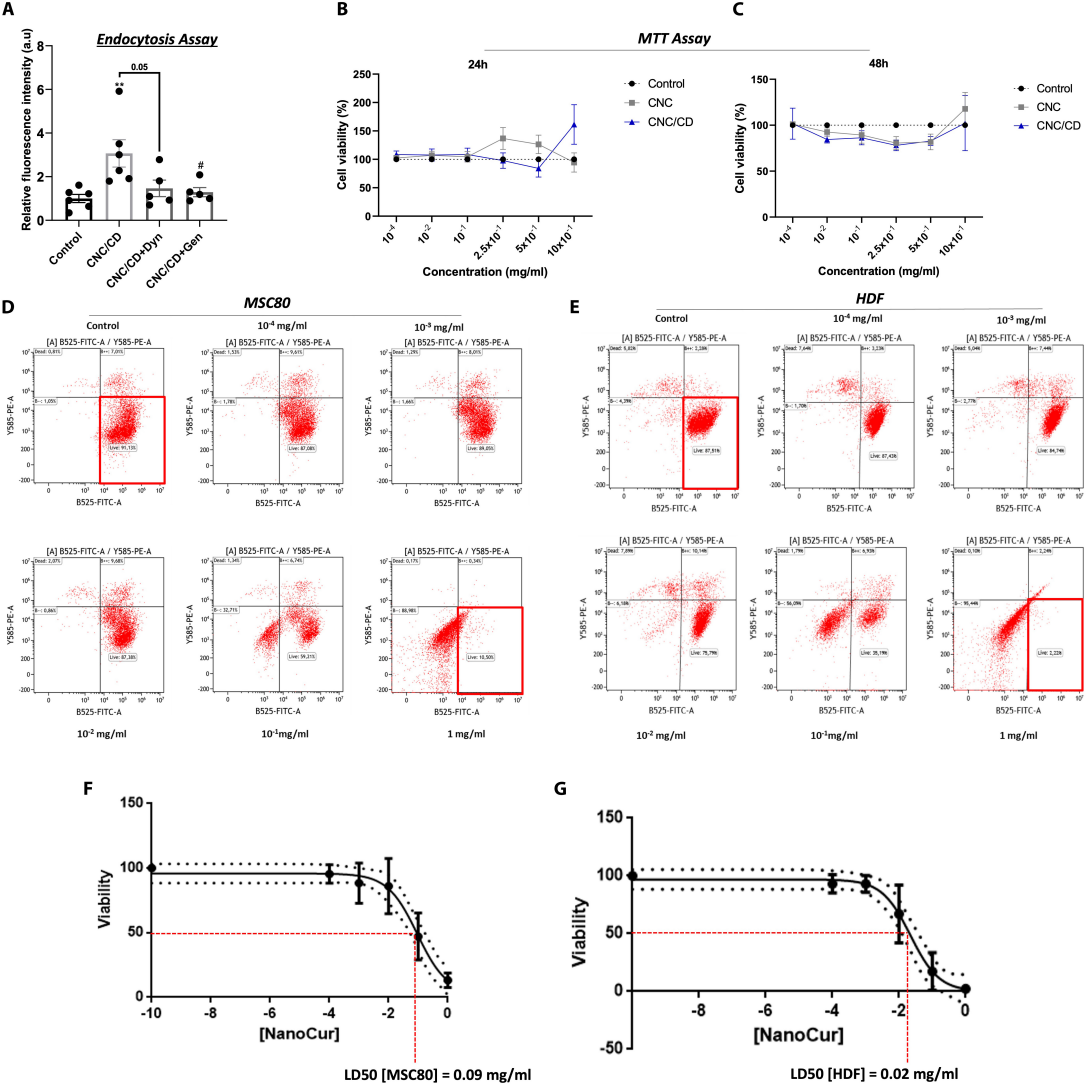
Assessment of the mechanism of entry and potential cellular toxicity of NanoCur in vitro. Fluorescence intensity of CW in (A) MSC80 cells treated with both CD/CNCs and CW in the presence or absence of endocytosis inhibitors dynasore (Dyn) and genistein (Gen). MTT assays were also performed on MSC80 cells treated with an increasing concentration of either CNC or CNC/CD ranging from 10^-4^ to 1 mg/ml for 24 h (B) and 48 h (C). Cell viability measured in (D) MSC80 and (E) HDF exposed to 24 h of increasing dose of NanoCur ranging from 10^-4^ to 1 mg/ml through flow cytometry using live/dead assay with LD50 determined using GraphPad Prism software (F and G). Results are represented as mean ± SEM of 4 to 6 independent experiments. Statistical test performed using 1-way ANOVA followed by Tukey’s post hoc test. ^**^P < 0.01 versus Ctr; ****P < 0.0001 versus Ctr; #*P* < 0.05 versus CNC/CD; ^##^*P* < 0.01 versus CNC.

It has been previously reported that the CNC/CD vector can aid curcumin entry into cells, being more effective than curcumin alone [17]. We thus hypothesized that the cellulose nanocrystals acting as excipients would help curcumin reach the cellular target. Therefore, CNC/CD vectors were conjugated to CW and intracellular fluorescence intensity of the CNC/CD/CW complex was measured. Results showed a significant increase in CW fluorescence in MSC80 incubated with CNC/CD/CW complex compared to control cells exposed to CW alone. Interestingly, cotreatment of cells with the endocytosis inhibitors dynasore or genistein led to a significant decrease in CW fluorescence, strongly suggesting that the CNC/CD complex delivers curcumin into the cell via endocytosis (Fig. 6A).

The safety of the different components of the NanoCur complex was tested at the cellular level. Treatment of MSC80 with increasing concentrations of either CNC or CNC combined with CD (CNC/CD) did not result in significant cell death, neither after 24- nor 48-h exposure to either CNC or CNC/CD (Fig. 6B and C). To check for toxicity of curcumin in NanoCur, MSC80 cells and HDF were also treated with increasing concentrations of the curcumin-bearing complex for 24 h and cell survival and LD50 were determined. Curcumin inside the nanocomplex also showed a good safety threshold, with cellular toxicity only observed at very high concentrations (Fig. 6D and E). Furthermore, the LD50 of NanoCur in MSC80 cells was approximately 0.09 and 0.02 mg/ml in HDF (Fig. 6F and G); with these concentrations being significantly above the reported effective concentrations of NanoCur [18].

**Fig. 7.**
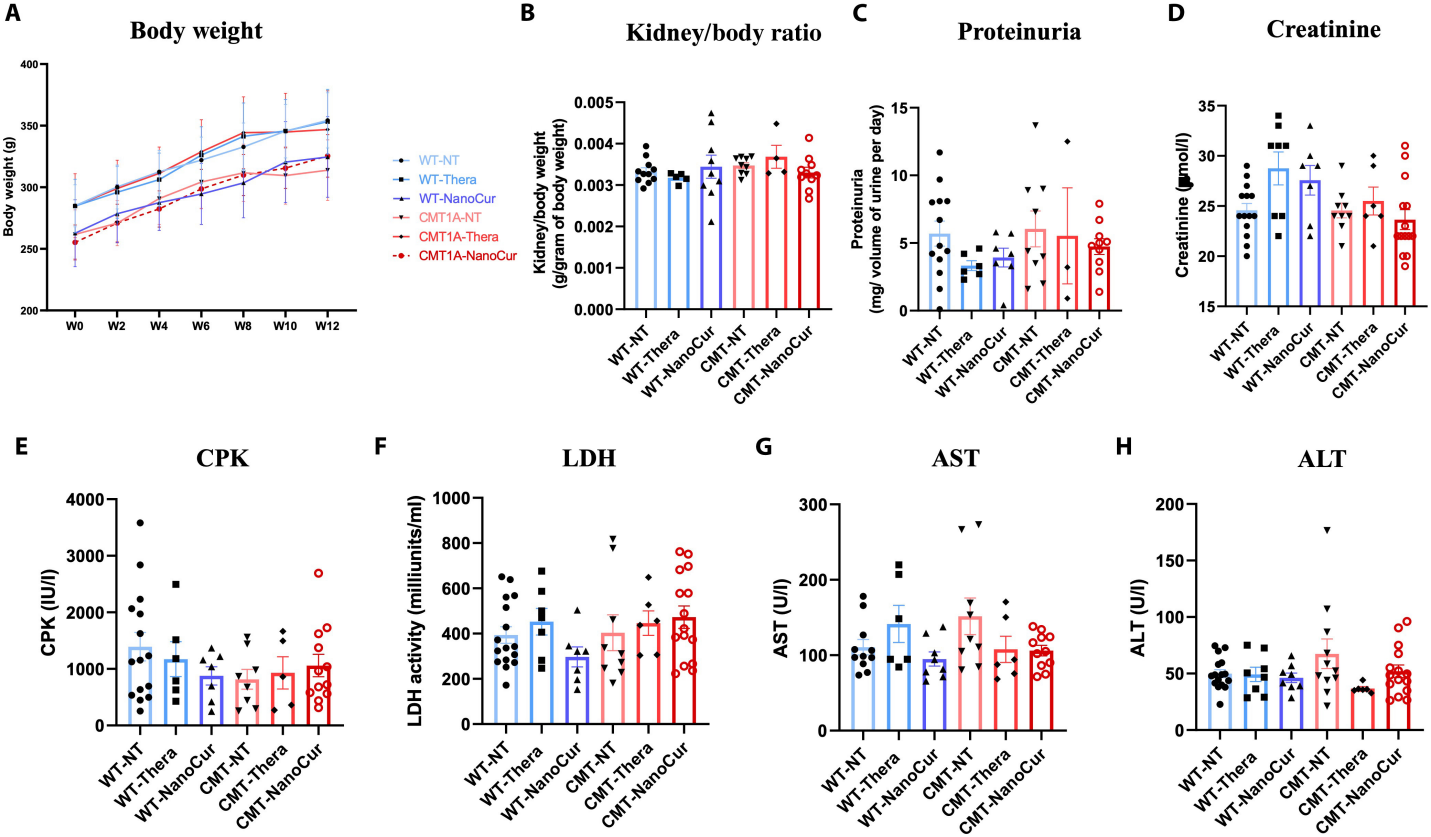
Investigation of the systemic toxicity of NanoCur and Thera following daily administration in rats over 12 weeks. NanoCur and Thera safety were assessed by (A) comparison of body weight, (B) kidney/body weight ratio as an index of kidney hypertrophy, (C) proteinuria, (D) blood creatinine, (E) CPK level, (F) LDH activity and liver, (G) AST, and (H) ALT levels. Results are represented as mean ± SEM. For (A), statistical analysis was performed using 2-way ANOVA followed by Tukey’s post hoc, while 1-way ANOVA followed by Tukey’s post hoc test was performed for the others (*n* = 11 to 18 WT-NT, *n* = 5 to 9 WT-Thera, *n* = 7 to 9 WT-NanoCur, *n* = 8 to 13 CMT1A-NT, *n* = 3 to 7 CMT1A-Thera and *n* = 9 to 16 CMT1A-NanoCur), ; *: *P* < 0.05; **; *P* < 0.01; ***: *P* < 0.001; ****: *P* < 0.0001; ^*^ns: nonsignificant.

It is not known if long term treatment with NanoCur could have unwanted effects on normal physiological function. We assessed the effect of treatment of rats with either saline, NanoCur or Thera on a systemic level. There were no deaths in any of the treatment groups throughout the study. Body weight was monitored throughout the treatment period and no significant difference was recorded among the treated animals compared to their control littermates (Fig. 7A). Nephrotoxicity was first assessed by measuring kidney hypertrophy, proteinuria, and blood creatinine. Interestingly, kidney/body weight ratio, urine protein concentration and blood creatinine were similar to controls (Fig. 7B to D)*.* Liver and tissue damage were also assessed using plasma markers of injury including CPK, LDH, ALT, and AST, which all showed values that were comparable to controls (Fig. 7E to H). Overall, these results suggest no nephrotoxicity or hepatotoxicity due to extended exposure, confirming the safety of a prolonged administration of NanoCur, and potentially of Thera, in vivo.

## Discussion

In the absence of effective therapies, new strategies are needed for the treatment of neurodegenerative diseases and nanosystems are a candidate therapy that could fill the gap in this area [28]. In the case of CMT1A, only one potential treatment, PXT3003, has progressed from the preclinical phase and is being currently undergoing an extensive phase 3 trial [29–31]. Despite the potential of PXT3003 (a combination of baclofen, naltrexone, and sorbitol), in which the therapeutic effect is related to downregulation of PMP22, an effect that is similarly observed when PMP22-specific small interfering ribonucleic acid formulations are used [1], we believe our novel NanoCur treatment may have a broader mechanism of action, which might be beneficial to several peripheral neuropathies in addition to CMT1A. In fact, the strategies involving the reduction of PMP22 levels are potentially destined to be of limited translational potential, essentially due to the variation in PMP22 expression among CMT patients, thus requiring a very precise approach that must be tailored to each patient. This will require a sufficient reduction in PMP22 to be translated at the clinical level to avoid the induction of another PMP22-associated disease, such as hereditary neuropathy with pressure palsies. In terms of mechanism, our previous study emphasized the role of NanoCur in alleviating oxidative and ER stress mechanisms in peripheral nerves of CMT1A rodents [18]. The data presented in the current study also support an anti-inflammatory potential of the NanoCur, providing a triple hit mechanism for relief of nerve injury in CMT1A. More importantly, these 3 mechanisms of injury are thought to overlap, and tend to be activated in a loop that ultimately exacerbates the injury in multiple organ systems and pathologies [32,33]. Interestingly, curcumin, which has multiple biological properties, has previously been studied in humans as a candidate treatment for several of these pathologies, with multiple clinical trials performed to study its effect [34]. Unfortunately, most of these trials failed, essentially due to the low stability and bioavailability of curcumin formulations that were used. In this context, our aim was to develop a novel curcumin formulation that ensures that curcumin can reach the target organ, while avoiding systemic toxicity. Moreover, we aimed at validating NanoCur as a novel therapy for CMT1A and potentially for other neuropathies such as diabetic and traumatic peripheral neuropathies.

The positive outcome of the initial study on animal models with early-stage disease [18] led us to investigate the potency of NanoCur in a more advanced stage of the disease. After 12 weeks of chronic treatment of 3-month-old male and female CMT1A rats, NanoCur was successful in significantly reversing the course of the disease and treating the neurological symptoms. In addition, NanoCur was compared to a commercially available curcumin formulation, Thera. Using similar doses and route of administration, we observed a striking improvement in neurofunctional parameters as shown by the significant increase in MNCV of NanoCur-treated animals compared to nontreated controls. This was accompanied by an overall incremental improvement in sensorimotor coordination and neuromuscular force (Fig. S2). A significantly lesser effect was seen in Thera-treated rats. Despite the overall positive outcome of the study, we must keep in mind the heterogeneity between animals with regard to their initial phenotype and the final treatment outcome, similar to what is observed in clinical studies on CMT patients. For example, CMT1A rats differ in symptom intensity between sexes as well as between littermates. In fact, the severity of the phenotype varied across the batches of experimental animals used for this study, with some animals impacted more than others. The causes of this variability are unknown, though epigenetic and environmental modifications have been suggested [35,36]. This phenotypic variation provides us with challenges when analysing data and could be deceptive when assessing the effectiveness of possible therapies, leading to the need for high sample sizes to compensate for variation. In terms of histology, the demyelinating profile seen in CMT1A rat sciatic nerves was improved by NanoCur, where treatment was able to re-establish an almost normal myelin phenotype, which translated functionally into an increase in the MNCV in the NanoCur-treated group. These findings are in agreement with previous studies that reported that curcumin alone or introduced using nano-vectors improved myelination in many peripheral and central disease models [37].

Validation of novel therapeutic compounds requires studies of their potency in different physiological models, where drug metabolism, among other factors, can affect the therapeutic benefit of the studied compound [38]. For this purpose, a preliminary study was conducted on C61 mice, a model of CMT1A which has a pathology that closely resembles that seen in humans [23]. The current data suggest that NanoCur is remarkably effective in alleviating motor symptoms in hemizygous mice, and more importantly in homozygous mice. Macrophage activation was confirmed in the transgenic mouse model with a relative reduction seen in response to NanoCur administration. These results suggest that NanoCur may provide a solid therapeutic platform for CMT1A treatment across the spectrum of the disease, from moderate to more severe forms.

At a molecular level, we investigated 3 mechanistic targets of NanoCur; effects on oxidative stress, ER stress, and inflammation. In our earlier study, we showed that the oxidative status was enhanced in the sciatic nerve concomitant to increased levels of antioxidant enzymes including PRDX1, PRDX5, and TRX1 in response to NanoCur administration. This effect appeared to be replicated in the older animals used in the current study with other antioxidant enzymes appearing to be involved in this response. Specifically, superoxide dismutase 1 protein, known to play a major role in the antioxidant response and to be involved in the development of neurodegenerative diseases such as amyotrophic lateral sclerosis (ALS) [39,40], was found to be significantly upregulated in treated animals. Regarding ER stress, the level of calnexin, which was initially shown to increase in response to NanoCur treatment, then showed a tendency to decline following the 12-week-treatment regimen (data not shown). Indeed, Hammond et al. demonstrated that, the chaperone protein calnexin, which serves as a crucial monitor of quality control and ensures proper folding of proteins intended for the plasma membrane or extracellular secretion [41], selectively folds glycoproteins like PMP22 [42]. Its elevation in CMT1A may occur to stop misfolding of PMP22. Hence, the decrease in its level in the NanoCur-treated group might indicate an improvement in ER stress and a reduced need for corrective proteins as the general ER condition becomes less severe. In terms of inflammation, neuroinflammation has been suggested to contribute to the pathology of both demyelinating and axonal CMT [27]. In this context, we hypothesized that macrophages could play an important role in the propagation of nerve injury. Although being crucial to nerve, and specifically Schwann cell, homeostasis [43], their chronic activation might play a detrimental role at the site of injury [25]. Our data support an anti-inflammatory role for NanoCur through a reduction in macrophage recruitment to the sciatic nerve as shown by decreased ED-1 (CD68) staining in the treated CMT1A rats. Furthermore, the protein level of MIF was increased while the proinflammatory protein myeloperoxidase was decreased in response to treatment. Altogether, these data point to the establishment of a lower-grade inflammatory milieu in CMT1A affected animals as a result of NanoCur injection. However, macrophage involvement requires further investigation as well as the dissection of the interaction with other molecular pathways including oxidative and ER stress mechanisms. Such knowledge will provide more solid evidence concerning the primary therapeutic target of NanoCur and assist in validating NanoCur as a treatment for improving nerve injury in CMT1A.

To conclude, curcumin has previously been found to have a number of positive health benefits, including anti-inflammatory, anti-microbial, anti-carcinogenic, and hypoglycaemic effects, combined with very low toxicity, even at high doses, in both clinical and preclinical studies [44,45]. Unfortunately, the use of this compound is challenging due to its poor pharmacological properties related to low bioavailability, resulting in its therapeutic effects being very limited. For this reason, our CNC/CD vector was developed, an encapsulation method that drastically increased curcumin bioavailability and thus the capacity to exert its therapeutic function. Indeed, the increased bioavailability of curcumin was supported by higher plasma concentration that were observed upon the injection of a single dose of NanoCur, but not Thera, with a peak value around 2 h posttreatment (Fig. S3). However, the present study lacks evidence of NanoCur readily crossing the blood-nerve barrier. In this regard, the reported ability of cellulose nanoparticles to cross the blood–brain barrier appears promising in terms of evidence for potential access to peripheral nerves [46]. We thus believe that NanoCur is an interesting candidate treatment for CMT1A as well as for other neuropathies with similar underlying mechanisms of injury. In this context, more complex studies will certainly be required to fully understand the pharmacokinetic profile (absorption, distribution, metabolism, and elimination) of NanoCur and, consequently, the dose and mode of administration required for treatment of CMT1A in humans.

## Data Availability

The data that support the findings of this study are available from the funding agency “*AFM-Telethon”* but restrictions apply to the availability of these data, which were used under license for the current study, and so are not publicly available. Data are however available from the authors upon reasonable request and with permission of *AFM-Telethon.*
