# Supplementary material for: Improvement of Charcot-Marie-Tooth Phenotype with a Nanocomplex Treatment in Two Transgenic Models of CMT1A
Source: Biomater Res. 2024 Mar 28;28:0009. doi: 10.34133/bmr.0009 (PMC10981932; doi:10.34133/bmr.0009)
Supplement: Supplementary 1 — Figs. S1 to S3 [file bmr.0009.f1.zip › Supplementary Materials.docx]

Supplementary Materials

Additional file: Fig. S1. Original western blot membranes along with the stain free membrane captures for the (A) macrophage migration inhibition factor (MIF) and (B) myeloperoxidase antibodies, displayed in the original manuscript text. Fig. S2. Time-dependent follow up of the grip force of the WT and CMT rats starting at week 0 (W0) till W12 of treatment with either saline, Theracurmin or NanoCur. Results are represented as mean ± SEM. Statistical test was performed using Two-Way ANOVA followed by Tukey’s Post-hoc Test; *** P˂0.001 (n=18 WT-NT, n=9 WT-Thera, n=9 WT-NanoCur, n=13 CMT1A-NT, n=7 CMT1A-Thera & n=16 CMT1A-NanoCur). Fig. S3. Quantification of the plasma concentration of curcumin after 0, 1, 2, 4, 8 and 24 hours of intraperitoneal treatment of rats with 0.2 mg of curcumin/kg of body weight using either NanoCur or Theracumin. Quantification was performed by mass spectrometry at the BISCEm multi-thematic platform at the University of Limoges.

**Fig. S1.** Original western blot membranes along with the stain free membrane captures for the **(A)** macrophage migration inhibition factor (MIF) and **(B)** myeloperoxidase antibodies, displayed in the original manuscript text.

**Fig. S2.** Time-dependent follow up of the grip force of the WT and CMT rats starting at week 0 (W0) till W12 of treatment with either saline, Theracurmin or NanoCur. Results are represented as mean ± SEM. Statistical test was performed using Two-Way ANOVA followed by Tukey’s Post-hoc Test; *** P˂0.001 (n=18 WT-NT, n=9 WT-Thera, n=9 WT-NanoCur, n=13 CMT1A-NT, n=7 CMT1A-Thera & n=16 CMT1A-NanoCur)


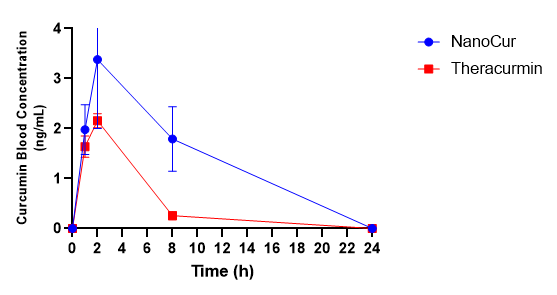


**Fig. S3.** Quantification of the plasma concentration of curcumin after 0, 1, 2, 4, 8 and 24 hours of intraperitoneal treatment of rats with 0.2 mg of curcumin/kg of body weight using either NanoCur or Theracumin. Quantification was performed by mass spectrometry at the BISCEm multi-thematic platform at the University of Limoges.
