# Supplementary figures and images for: Improvement of Charcot-Marie-Tooth Phenotype with a Nanocomplex Treatment in Two Transgenic Models of CMT1A
Source: Biomater Res. 2024 Mar 28;28:0009. doi: 10.34133/bmr.0009 (PMC10981932; doi:10.34133/bmr.0009)

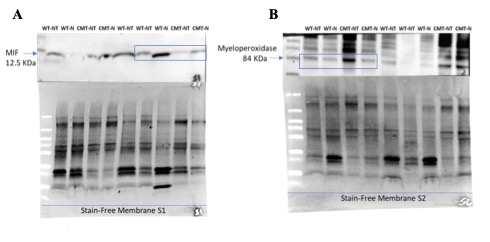

Supplement: Supplementary 1 — Figs. S1 to S3 [file bmr.0009.f1.zip › Figure S1-BMR-D-23-00040.jpg]

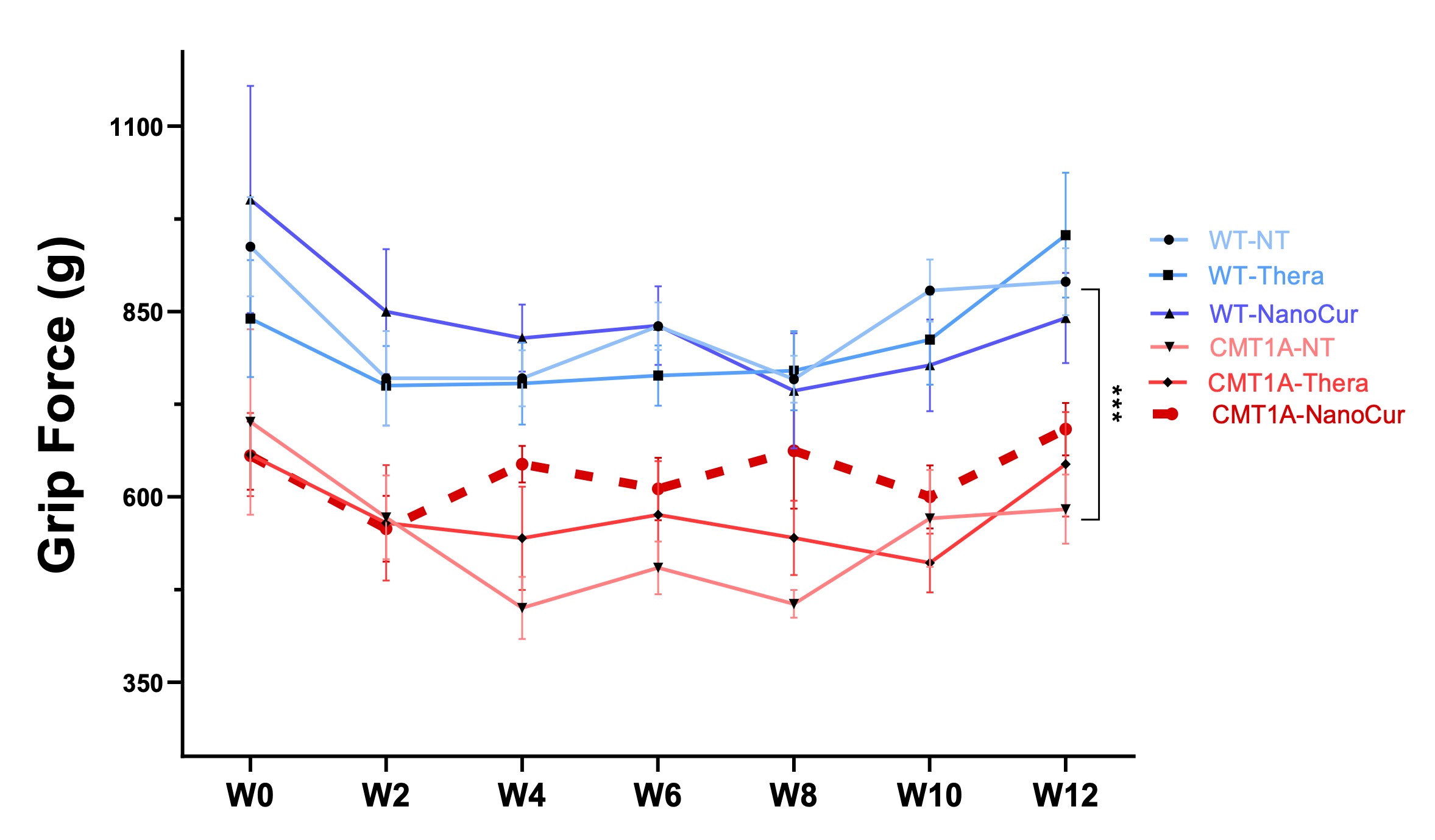

Supplement: Supplementary 1 — Figs. S1 to S3 [file bmr.0009.f1.zip › Figure S2-BMR-D-23-00040.jpg]

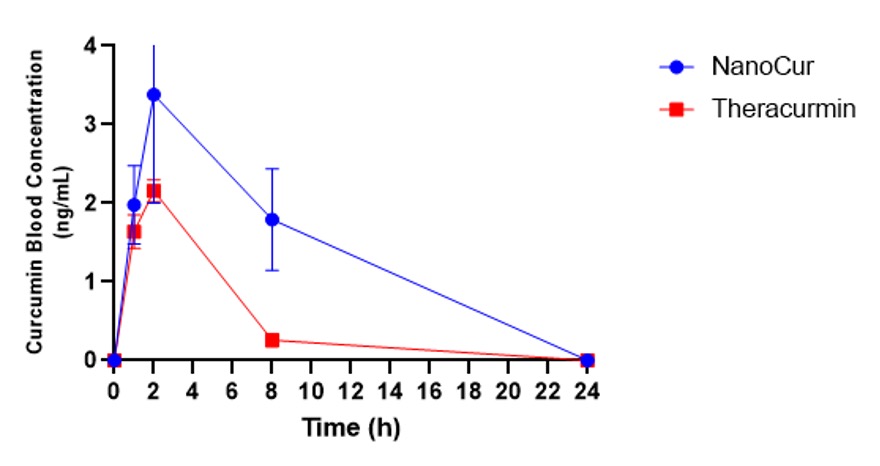

Supplement: Supplementary 1 — Figs. S1 to S3 [file bmr.0009.f1.zip › Figure S3-BMR-D-23-00040.jpg]
